# An analysis of the effect of agriculture subsidies on technical efficiency: Evidence from rapeseed production in China

**DOI:** 10.1016/j.heliyon.2024.e33819

**Published:** 2024-06-28

**Authors:** Fuxing Liu, Muhammad Aamir Shahzad, Zhongchao Feng, Lianfen Wang, Juan He

**Affiliations:** a College of Economics & Management, Huazhong Agricultural University, Wuhan, 430070, China; b Hubei Rural Development Research Center, Wuhan, 430070, China; c School of Economics and Trade, Hunan University, Shijiachong, Yuelu District, Changsha City, Hunan Province, 410079, China

**Keywords:** Agricultural subsidies, Production technology efficiency, Factor input, Stochastic frontier production function, Rapeseed

## Abstract

This study was conducted to examine the effects of agricultural subsidies on the technical efficiency of agricultural production technology and on factor input. It utilized a random frontier production function, instrumental variable method, and threshold regression model. The data used for this analysis consisted of 609 field yield measurements from the National Rapeseed Industry Technology System in 2020. The findings indicate that agricultural subsidies have a substantial impacts and it increases the technical efficiency of production process. Specifically, these subsidies encourage the use of land resources while inhibiting the use of chemical fertilizers. However, this does not have a significant effect on the utilization of labor and capital resources. Furthermore, the impact of agricultural subsidies on production technology efficiency varies depending on the scale of the farming operation. The subsidies significantly enhance the production technology efficiency of farmers with a business scale of less than 0.67 ha, but do not significantly improve the production technology efficiency of farmers with a business scale exceeding 0.67 ha. To optimize the effectiveness of agricultural subsidy policy, three methods and recommendations are proposed: increasing the overall amount of subsidies, expanding and diversifying the types of subsidies, and refining the process of disbursing subsidies.

## 1 Introduction

Agricultural subsidies are typical policy strategies used by countries around the world to prevent the “Ricardian Trap” of industry feeding agriculture during the economic development process. Since 2004, China has steadily eliminated agricultural tariffs and built an agricultural subsidy scheme [1,2]. As domestic chemical fertilizer and fuel prices rose due to the influence of international pricing in 2006, the Chinese agricultural subsidy program was expanded to include “Aggregate input subsidy” [3]. Chinese agricultural subsidies totaled 164.3 billion rmb (ren min bi- China currency) in 2012 [4]. The agricultural subsidy policy was modified in 2015 by the Ministries of Finance and Agriculture and Ministry of Agriculture and Rural Affairs of China [5]. The government has set out 120.485 billion rmb for year 2020. The Chinese government's agricultural output subsidies are consistently increasing (Ministry of Finance, 2020) [6,7].

The primary goal of agricultural subsidies is to increase total production capacity and preserve national food security [[8], [9], [10]]. Agricultural output is directly linked to farmer factors allocation, and technical efficiency is the centralized expression of total agricultural production potential [11]. The studies by Adom et al., 2020 and Ogundari 2014 revealed that technical efficiency in agriculture is critical for productivity increase and poverty reduction [12,13].

Previous research has examined the impact of agricultural subsidies on the input of production factors. For example, some research has found that agricultural subsidies increase planting area, motivate farmers to plant more crops [14,15]. Several studies in the literature have found that agricultural subsidies reduce the use of pesticides and fertilizers [16,17], but it increases agricultural employment, and agricultural machinery usage [[18], [19], [20], [21]]. The research by Bojnec et al., 2013 revealed that Slovenian farm size positively affects technological efficiency and small farms are less efficient technically but more profitable allocatively. Small farms in Slovenia may survive due to large subsidies [22]. The Barath et al., 2020 found to have no significant effect of subsidies on either TFP or on its components [23].

Theoretical findings on the subsidy-efficiency relationship are equivocal [24]. According to some research, agricultural subsidies improve technical efficiency [25,26]. Some research argues that agricultural subsidies have a negligible impact on agricultural output, even causes a decrease in technical efficiency [27,28]. Subsidies may reduce farmers’ efforts or change their risk attitudes, resulting in a decrease in technical efficiency [[29], [30], [31]]. However, to the best of our knowledge, there is little relevant evidence on the impact of agricultural subsidies on the technical efficiency of rapeseed oil in China.

Rapeseed is one of China's most important oil crops. According to USDA data, the world harvested 52.047 million acres of rapeseed in 2020, with China accounting for 19.08 % of that amount worldwide and placing third globally. However, China imports a lot of rapeseed and rapeseed oil. In 2020, China was the world's second-largest importer of rapeseed and rapeseed oil, with imports reaching 15.043 million tones and 5.166 million tones (USDA). In China, there is still a substantial supply and demand imbalance in rapeseed production. As a result, improving the technical efficiency of rapeseed production in China is critical not only for ensuring the Chinese people's access to edible vegetable oil, but also for ensuring global food security and eliminating poverty. Little literature exists to answer these two questions. Can agricultural subsidies improve technical efficiency in rapeseed oil in China? And what is the impact of various factor inputs? To answer these questions, this study aims to investigate the impact of agricultural subsidies on technical efficiency and their impact on factor inputs.

The contributions of this study are mainly as follows: first, we used 609 field yield measurement data from 27 experimental stations in 14 provinces of the National Rapeseed Industry Technology System in China. It enriched the literature on agricultural subsidies and technical efficiency. Second, the stochastic frontier function model was employed by introducing an interaction term between agricultural subsidies and factor inputs, and the effects of agricultural subsidies on factor inputs and technical efficiency are analyzed simultaneously. Third, we used a threshold regression model to analyze the effect of agricultural subsidies on the technical efficiency of rapeseed oil in different planting areas and found a threshold value of 0.67ha.

## 2 Material and methods

### 2.1 Econometric model

The stochastic frontier production function model was first proposed independently by Aigner et al., 1977 and Meeusen et al., 1977 respectively [32,33], and subsequently a number of scholars have made different assumptions about the form of the distribution of technically inefficient terms, which have been widely used in empirical studies [34]. The stochastic frontier production function indicates that there exists a deterministic production frontier indicating the optimal output for a given technology and input level in a given period (refer to equation (1)). The general form can be expressed as follows:

(1) $$\ln\;y_{i}=f\left(x_{i};\beta \right)+v_{i}-u_{i}$$

Whereas in equation (1), $\ln\;y_{i}$ is the actual output; $f\left(x_{i};\beta \right)$ is the production frontier; $x_{i}$ is the vector of production factor inputs; $v_{i}$ is the zero-mean random noise term, which is assumed to follow a normal distribution; $u_{i}$ is the technical inefficiency term between 0 and 1. If $u_{i}\geq 0$, this indicates the presence of technical inefficiency, expressing that the actual output is smaller than the optimal output of the production frontier. Generally, the ground distribution takes the form of half-normal, exponential, gamma distribution and truncated normal distributions. In this paper, we assume that the distribution obeys the half-normal distribution, $u_{i}∼N^{+}\left(0,\sigma_{u}^{2}\right)$, and also assume that $v_{i}$ and $u_{i}$ is independently identically distribution. According to Battese et al., 1995, estimating the exogenous variables that influence technical inefficiency can be described as the following: $\sigma_{u}^{2}=\exp\left(z_{i};\omega \right)$. Where $z_{i}$ is the factor that may affect the inefficiency term, and ω is the estimated coefficient of interest [35].

It is necessary to make assumptions on the form of the production function before examining how agricultural subsidies affect technical production efficiency. It satisfies the need of the research because the transcendental logarithmic production function is more adaptable than the conventional Cobb-Douglas(C-D) production function which permits output and substitution elasticities to vary with factor inputs. We found that existing literature generally ignores the role of agricultural subsidies on factor inputs while studying the impact of agricultural subsidies on agricultural production technology efficiency. When constructing the model, they included agricultural subsidies as an exogenous variable in the model. To solve this issue, we referred to the research done by Roll 2019 [36], we use agricultural subsidies as an input variable, the following equation (2) presents *trans*-log function model which is constructed in this paper:

$$\ln\;y_{i}=\beta_{0}+\beta_{land}lnland+\beta_{labor}lnlabor+\beta_{capital}lncapital+\beta_{fertilizer}lnfertilizer+$$

$$0.5\sum_{k}\sum_{l}\beta_{kl}\;\ln\;x_{k}*\ln\;x_{l}+\beta_{subsidy}lnsubsidy+0.5\beta_{subsidy2}{\left(lnsubsidy\right)}^{2}+$$

$$\beta_{subsidy*land}lnsubsidy*land+\beta_{subsidy*labor}lnsubsidy*labor+$$

(2) $$\beta_{subsidy*capital}lnsubsidy*capital+\beta_{subsidy*fertilizer}lnsubsidy*fertilizer+v_{i}-u_{i}$$

where *y* is the output variable; *land* is the land factor input; *labor* is the labor factor input; *capital* is the capital input; *fertilizer* is the fertilizer factor input; and *subsidy* is the agricultural subsidy. To simplify the model construction process, the two-by-two interaction terms between the four factor input variables of land, labor, fertilizer, and capital are denoted by equation (3) following:

(3) $$0.5\sum_{k}\sum_{l}\beta_{kl}\;\ln\;x_{k}*\ln\;x_{l}$$

Additionally in equation (4), the output elasticity of agricultural subsidies, or the partial derivative of agricultural subsidies on the output variable, can be used to indicate how agricultural subsidies affect the output variable:

$$\varepsilon_{subsidy}=\frac{{\partial lny}_{i}}{{\partial lnsubsidy}_{i}}=\beta_{subsidy}+\beta_{subsidy}lnsubsidy+\beta_{subsidy*land}lnland+$$

(4) $$\beta_{subsidy*labor}lnlabor+\beta_{subsidy*capital}lncapital+\beta_{subsidy*fertilizer}lnfertilizer$$

If $\varepsilon_{subsidy}>0$, then agricultural subsidies are likely to have a positive impact on output variables. Otherwise, they are likely to have a negative impact. The output elasticity of agricultural factor of production input variables with regard to agricultural subsidies can be determined by considering the partial derivatives independently in order to examine the impact of agricultural subsidies on each component of production input [refer to equation (5), equation (6), equation (7), and equation (8)]:

(5) $$\frac{{\partial \varepsilon }_{subsidy}}{\partial land}=\beta_{subsidy*land}$$

(6) $$\frac{{\partial \varepsilon }_{subsidy}}{\partial labor}=\beta_{subsidy*labor}$$

(7) $$\frac{{\partial \varepsilon }_{subsidy}}{\partial capital}=\beta_{subsidy*capital}$$

(8) $$\frac{{\partial \varepsilon }_{subsidy}}{\partial fertilizer}=\beta_{subsidy*fertilizer}$$

If $\beta_{subsidy*land}$, $\beta_{subsidy*labor}$, $\beta_{subsidy*capital}$, $\beta_{subsidy*fertilizer}>0$, It shows that agricultural subsidies increase the input costs for each production factor. Otherwise, it suggests that agricultural subsidies decrease each input element. This study makes the case that it is unknown which way agricultural subsidies will affect agricultural factor inputs.

To analyze the effect of agricultural subsidies on the technical efficiency of agricultural production, a linear equation (9) on the technical inefficiency term of agricultural production is constructed as follows:

(9) $$\sigma_{u}^{2}=\lambda_{0}+\lambda_{subsidy}lnsubsidy+\lambda_{i}X_{i}$$

Where $\lambda_{subsidy}$ represents the direction of the effect of agricultural subsidies on technical inefficiency. If $\lambda_{subsidy}>0$, it indicates that agricultural subsidies have a positive effect on the technical inefficiency term of production, that is, agricultural subsidies reduce the technical efficiency. Otherwise, it indicates that agricultural subsidies had a negative effect on the technically inefficient term of production, that is, agricultural subsidies increased the technical efficiency of production. Accordingly, this paper argues that the direction of the effect of agricultural subsidies on the technical efficiency of agricultural production is uncertain.

### 2.2 Variables and descriptive analysis

There are three sets of variables used in this study: (1) technical efficiency, (2) agricultural subsidies, and (3) other control variables.

Technical efficiency is the percentage of the minimum cost required to produce a given quantity of a product at a given ratio of factor inputs to the actual cost, given constant production technology and market prices [37], it reflects the ability to maximize output for a given set of inputs [38]. Technical efficiency is calculated from the stochastic frontier production function. In terms of the stochastic frontier production function, the dependent variable is rapeseed oil yield. Meanwhile, the independent variables include the input of labor, fertilizer and capital.

Agricultural subsidies, the variable of interest, is measured by the total amount of subsidies from the government. It includes scale management subsidies, machinery subsidies, arable land conservation subsidies, and other subsidies.

Other control variables include three groups. The first group describes the individual characteristics, such as gender, age, education level, and health status. The second group describes the household characteristics, such as number of family members, number of farm workers, and transportation conditions; The third group describes the production characteristics, such as cooperatives, agricultural technology training, mechanization degree, and agricultural information access. The descriptive statistics of the variables is shown in Table 1.

**Table 1:** Summary statistics for the study's variables.

Symbol	Variables definition	Mean	SD
Yield	Total rapeseed production (KG)	3813.51	13607.81
Land	Rapeseed sown area (HA)	2.09	6.91
Labor	Self-employed and employment costs (rmb)	6247.44	20179.02
Fertilizer	Fertilizer usage (KG)	458.61	1774.37
Capital	Material and service charges (rmb)	4953.75	18023.98
Subsidy	Agricultural subsidies (rmb)	11122.03	72718.20
Gender	Male = 1, Female = 0	0.92	0.26
Age	Actual age (years)	58.16	9.61
Education	Illiteracy = 1, Primary = 2, Junior = 3, Senior = 4, University = 5	2.78	0.77
Health	Poor = 0, Same = 1, Good = 2	1.42	0.57
Family	Number of family population	4.90	1.84
Farming	Number of farmers at home	2.10	0.86
Traffic	Distance to agricultural service station (KM)	4.24	3.57
Cooperative	Whether to join the cooperative (Yes = 1, No = 0)	0.27	0.45
Training	Whether to participate in training (Yes = 1, No = 0)	2.68	4.41
Machine	Ratio of machine tillage and sown area	0.18	0.38
Information	Whether to use WeChat (Yes = 1, No = 0)	0.85	0.36

### 2.3 Data

The data collected are all from the National Rapeseed Oil Industrial Technology System's 2020 field yield measurement survey data. This data was used for two main reasons. First, the field yield survey included 27 experimental stations spanning 14 major rapeseed oil-producing provinces in China (These 14 provinces include: Anhui, Guangxi, Guizhou, Henan, Hubei, Hunan, Jiangsu, Jiangxi, Shaanxi, Shanghai, Sichuan, Yunnan, Zhejiang, and Chongqing), which accounted for 91.54 % of total national rapeseed oil production. Second, at each experimental station, full-time employees collected field yield survey data. The full-time technicians at the experimental stations have vast experience working on the front lines of the rapeseed industry, have in-depth knowledge of the complete rapeseed cultivation process. They also possess substantial survey data expertise.

The datasets were collected by constructing fixed observation sites across the country by using the technique of multi-stage random sampling. The specific data generation procedure was as follows: initially, three counties from each experimental station were chosen at random, followed by three villages and three rapeseed oil growers in each village. This meant that each experimental station required at least 27 rapeseed oil growers. Second, rapeseed growers were interviewed one-on-one in person, and the yield was measured by full-time technicians from adjacent experimental stations. Following that, questionnaires were filled out in accordance with the actual yield measurement. A total of 815 filled questionnaires were filled and returned for this field yield study. We analyzed and screened the data continuously to ensure its quality before opting to preserve 609 valid questionnaires after deleting outliers and missing entries. It is worth noting that due to limited funding and personal abilities, the data used is cross sectional for a single time period 2021.

The National Compilation of Information on Costs and Returns of Agricultural Products 2021 was used to validate the input-output data. Rapeseed yield was 2195.48 kg/ha in 2020, according to the "National Compilation of Information on Costs and Benefits of Agricultural Products in 2021," labor input was 7833.90rmb/ha (1 US$ dollar = 6.45 rmb in 2021, 7.24rmb in 2024), discounted fertilizer application was 246.45 kg/ha, and material and service expenditures were 2074.65rmb/ha. The sample data showed that rapeseed yield was 2232.00 kg/ha, labor input was 7921.20rmb/ha, fertilizer application was 443.1 kg/ha in discounted form, and material and service costs were 2076.60rmb/ha. Comparative analysis revealed that the two data sets are largely consistent, as shown in Table 2, indicating the sample data's validity and representativeness.

**Table 2:** Comparison of national and sample rapeseed production in 2020.

Input/output	Official data	Sample data
Yield	2195.48 kg/ha	2232.00 kg/ha
Labor	7833.90 rmb/ha	7921.20 rmb/ha
Fertilizer	246.45 kg/ha	443.10 kg/ha
Material and service expenses	2074.65 rmb/ha	2076.60 rmb/ha

## 3 Empirical results of the study

### 3.1 Results of stochastic frontier production function

Table 3 shows the estimation results of the stochastic frontier production function. According to the findings of model (1), the coefficient of γ is 0.9340, which is close to 1, indicating that the technical inefficiency component dominates the composite disturbance term. Moreover, δu, δv and λ passes the T-test, and the Wald value also passed the test at the 1 % level. In nutshell, from the results of the test, the construction of the stochastic frontier production function model is valid and robust. Table 3 shows the estimation results of equations (5), (6), (7), (8) using model (1) without compensating for the technical inefficiency aspect. The coefficient βsubsidy*land is 0.0222 and significant at the 5 % statistical level, indicating that agricultural subsidies have a significant positive impact on land factor inputs. This corresponds to the findings of Shelef et al., 2016 [39]. Agricultural subsidies encourage farmers to extend their farming, which encourage the transfer of agricultural land and hence enhance land factor inputs [40]. The coefficient βsubsidy*fertilizer is −0.0145 and significant at the 1 % statistical level, indicating that agricultural subsidies have a significant negative impact on fertilizer input. This is consistent with the findings of Guo et al., 2021 [41]. This is because of the reason that as the transfer payments, agricultural subsidies can increase farmers' income while diminishing farmers’ incentive to increase production and income by excessive fertilizer application. Agricultural subsidies, on the other hand, reduce preventive fertilizer use by changing farmers' predicted returns and dispersing their agricultural risks, causing farmers' production decisions to be reduced. The coefficient βsubsidy*labor and βsubsidy*capital are both negative but statistically insignificant, indicating that agricultural subsidies have no significant effect on labor factor input or capital factor input. This may be due to insufficient subsidies for labor and capital factor inputs.

**Table 3:** The result of stochastic frontier production function for technical efficiency.

Variables	Model (1)	
	Coef.	SE
*β*_*land*_	−0.5200	(0.4842)
*β*_*labor*_	0.1156	(0.2458)
*β*_*fertilizer*_	1.2158***	(0.2891)
*β*_*capital*_	0.2703	(0.2494)
*β*_land*land_	−0.3742***	(0.1099)
*β*_land*labor_	0.0685	(0.0821)
*β*_land*fertilizer_	0.5312***	(0.1486)
*β*_land*capital_	0.1538	(0.0966)
*β*_labor*labor_	0.0374	(0.0281)
*β*_labor*fertilizer_	−0.0961***	(0.0362)
*β*_labor*capital_	−0.0615	(0.0536)
*β*_fertilizer*fertilizer_	−0.1458**	(0.0654)
*β*_fertilizer*capital_	−0.1730***	(0.0531)
*β*_capital*capital_	0.0392	(0.0387)
*β*_*subsidy*_	0.0673	(0.0517)
*β*_subsidy*subsidy_	0.0026	(0.0030)
*β*_subsidy*land_	0.0222**	(0.0092)
*β*_subsidy*labor_	−0.0026	(0.0052)
*β*_subsidy*fertilizer_	−0.0145***	(0.0048)
*β*_subsidy*capital_	−0.0063	(0.0068)
*β*_*0*_	3.0923**	(1.3256)
λ_*0*_	−1.2793***	(0.0916)
σ_*u*_	0.5275***	(0.0241)
σ_*v*_	0.1402***	(0.0154)
*Γ*	0.9340	
Observation	609	

Note: ***, **, * represent significant at the 1 %, 5 % and 10 % statistical levels, respectively, as follows.

### 3.2 Baseline regression results

There are generally two methods to estimate the stochastic frontier production function (SFA), one-step SFA method and two-step SFA method. This paper uses a two-step SFA method for analysis (shown in Table 4), which means that the technical efficiency is estimated, then regressed on the technical efficiency as the explanatory variables and on the factors affecting technical efficiency as the set of explanatory variables. This has the advantage of facilitating the use of the instrumental variables approach to reduce endogeneity. At the same time, in order to avoid the estimation bias brought about by the different assumptions on the distribution of technical efficiency in the “two-step SFA method”, this paper also adopts the “one-step SFA method” for the robustness test.

**Table 4:** Results regarding agricultural subsidies and technical efficiency using two-step SFA method.

Variables	Model (2)		Model (3)		Model (4)	
	Coef.	SE	Coef.	SE	Coef.	SE
Subsidy	0.0053***	(0.0021)	0.0060***	(0.0021)	0.0056***	(0.0021)
Gender			−0.0314	(0.0261)	−0.0341	(0.0260)
Age			0.0005	(0.0008)	0.0005	(0.0008)
education			−0.0320***	(0.0100)	−0.0347***	(0.0100)
Health			0.0264**	(0.0121)	0.0278**	(0.0121)
Family			−0.0103***	(0.0038)	−0.0092**	(0.0038)
Farming			0.0045	(0.0082)	0.0030	(0.0082)
Traffic			0.0046**	(0.0019)	0.0043**	(0.0019)
cooperative					0.0030	(0.0156)
training					0.0228	(0.0151)
machine					0.0328*	(0.0182)
information					−0.0322	(0.0195)
Constant	0.6622***	(0.0130)	0.7285***	(0.0738)	0.7452***	(0.0763)
Observation	609		609		609	

Since technical efficiency has non-negative values and takes values ranging from 0 to 1. Therefore, the restricted Tobit model was used to estimate the values of Eq. (8). The outcomes are displayed in Table 4. Model (2) controls only agricultural subsidies, model (3) adds individual characteristic variables, and model (4) adds production characteristic variables. From the results of the stepwise regression, it can be seen that whether controlling only agricultural subsidies or adding other control variables, the impact of agricultural subsidies on the technical efficiency is significantly positive at a statistical level of more than 1 %, which indicates that agricultural subsidies can significantly improve the technical efficiency.

According to the estimation results of model (4), the estimated coefficient of agricultural subsidy is 0.0056, significant at 1 % statistical level, which indicates that 1 % increase in agricultural subsidy increases the technical efficiency by about 0.56 %. This is basically consistent with the findings of Kumbhakar et al., 2010 [42]. There are three primary theories for this: First, agricultural subsidies can motivate farmers to produce through the income and anticipation impact, leading farmers to expand their production and operation scale, which is consistent with the previous section's finding that agricultural subsidies boost land factor inputs. Second, agricultural subsidies can help reduce farmers' marginal costs of production, causing farmers to adjust the structure of each factor input and adopt greener and lower-carbon way of production, which improves output level, which is consistent with the conclusion that agricultural subsidies reduce chemical fertilizer factor input. Third, agricultural subsidies can encourage farmers to adopt new and improved varieties, which leads to increased output.

### 3.3 Results of instrumental variables for removing endogeneity

The preceding analysis indicates that agricultural subsidies have a strong positive effect on agricultural production technical efficiency. Given the potential endogeneity problem of the baseline regression, this paper employs “the average value of agricultural subsidies received by rural households” and “the availability of agricultural-related qualification certificates” as agricultural subsidy instrumental variables. The valid instrumental variables must meet two requirements: first, they must be related to agricultural subsidies; and second, they must be relevant. Secondly exogeneity, or the instrumental variables, are not directly related to agricultural production's technological efficiency. On one hand, the “average value of agricultural subsidies of rural households” reflects the level of agricultural subsidies in the farmers' home area. Simultaneously, farm-related qualification certificates reflect local government assistance to farmers, and farmers who hold qualification certificates are often eligible for certain agricultural subsidies. Both are closely connected with agricultural subsidies for farmers. On the other hand, neither has a direct impact on farmers' technical efficiency of agricultural production. As a result, these two instrumental variables logically satisfy the conditions of correlation and exogeneity. (Table 5 summarizes the estimation results).

**Table 5:** Results regarding agricultural subsidies and technical efficiency using instrumental variables.

Variables	Model (5)	
	Coef.	SE
Subsidy	0.0049*	(0.0028)
Gender	−0.0344	(0.0259)
Age	0.0005	(0.0008)
Education	−0.0346***	(0.0104)
Health	0.0276**	(0.0127)
Family	−0.0092**	(0.0036)
Farming	0.0034	(0.0082)
Traffic	0.0043**	(0.0017)
Cooperative	0.0033	(0.0161)
Training	0.0236	(0.0155)
Machine	0.0331*	(0.0172)
Information	−0.0315*	(0.0191)
Constant	0.7479***	(0.0744)
KP-LM statistics value	153.458***	
Cragg-Donald Wald F statistics value	404.528***	
Hansen J statistics value	3.854	
Observations	609	

After applying the instrumental variables technique to address potential endogeneity, the estimation findings of model (5) reveal that agricultural subsidies considerably and positively affect technical efficiency at the 10 % statistical level. A 1 % increase in agricultural subsidies raises farm households' technical efficiency by around 0.49 %. The estimated outcomes of the instrumental variables must also pass a number of tests. The unidentifiable test (KP-LM statistic) and the weak instrumental variables (Cragg-Donald Wald F statistic) findings in Table 5 reveal that the instrument factors employed are not identifiable or weak instruments. An over-identification test was required due to the inclusion of two instrumental variables, and the findings of the over-identification test (Hansen J statistic) similarly indicated that there were no over-identification problems. When selected instrumental variables are considered collectively, they are more reasonable and fit the conditions of valid instrumental variables.

### 3.4 Results of robustness test

#### 3.4.1 One-step SFA

To test the robustness of the results, we report the estimation results using the one-step method, as shown in Table 6. Model (6) solely accounts for the key explanatory variables, model (7) adds another control variables to model (6). According to the estimation results of model (7), the estimated coefficient of agricultural subsidies is −0.0466, which means that agricultural subsidies have a significant negative impact on the term of technological inefficiency, indicating that a 1 % increase in agricultural subsidies increases the technical efficiency of agricultural output by around 4.6 %. This is higher than Kumbhakar et al., 2010 findings [42]. The reason may be that the use of the one-step method solves the estimation bias caused by different assumptions when using the two-step method [43]. Hence, from the results, after using the one-step method, the agricultural subsidy still contributed significantly to the improvement of technical efficiency of rapeseed oil in China.

**Table 6:** Results regarding agricultural subsidies and technical efficiency using one-step SFA.

Variables	Model (6)		Model (7)	
	Coef.	SE	Coef.	SE
*β*_*land*_	−0.5779	(0.4745)	−0.5674	(0.4503)
*β*_*labor*_	0.0750	(0.2389)	0.1273	(0.2260)
*β*_*fertilizer*_	1.2587***	(0.2824)	1.1280***	(0.2787)
*β*_*capital*_	0.3007	(0.2475)	0.4009*	(0.2194)
*β*_land*land_	−0.4018***	(0.1077)	−0.3652***	(0.1072)
*β*_land*labor_	0.0527	(0.0803)	0.0794	(0.0767)
*β*_land*fertilizer_	0.5571***	(0.1462)	0.4948***	(0.1445)
*β*_land*capital_	0.1879*	(0.0976)	0.1788*	(0.0941)
*β*_labor*labor_	0.0419	(0.0269)	0.0426	(0.0260)
*β*_labor*fertilizer_	−0.0958***	(0.0349)	−0.0903***	(0.0346)
*β*_labor*capital_	−0.0525	(0.0520)	−0.0800	(0.0490)
*β*_fertilizer*fertilizer_	−0.1483**	(0.0645)	−0.1332**	(0.0632)
*β*_fertilizer*capital_	−0.1887***	(0.0526)	−0.1630***	(0.0518)
*β*_capital*capital_	0.0283	(0.0391)	0.0204	(0.0372)
*β*_*subsidy*_	0.0561	(0.0522)	0.0693	(0.0511)
*β*_subsidy*subsidy_	0.0032	(0.0029)	0.0034	(0.0029)
*β*_subsidy*land_	0.0209**	(0.0092)	0.0233**	(0.0091)
*β*_subsidy*labor_	−0.0034	(0.0054)	−0.0046	(0.0052)
*β*_subsidy*fertilizer_	−0.0148***	(0.0050)	−0.0145***	(0.0048)
*β*_subsidy*capital_	−0.0045	(0.0068)	−0.0059	(0.0067)
*β*_*0*_	3.1392**	(1.2952)	2.8900**	(1.1859)
*Inefficiency*				
*subsidy*	−0.0472**	(0.0240)	−0.0466*	(0.0258)
*gender*			0.1441	(0.2667)
*Age*			−0.0013	(0.0081)
*education*			0.3856***	(0.0974)
*health*			−0.2473**	(0.1240)
*family*			0.1095***	(0.0413)
*farming*			−0.1439*	(0.0803)
*traffic*			−0.0264	(0.0235)
*cooperative*			0.0614	(0.1540)
*training*			−0.3001*	(0.1560)
*machine*			−0.4653**	(0.1927)
*information*			0.1653	(0.1967)
λ_*0*_	−1.0129***	(0.1580)	−1.9677**	(0.7672)
Observation	609		609	

#### 3.4.2 Sample adjustments

To test the robustness of the estimation results, we also adjusted the samples, and the findings are shown in Table 7. Model (8) is the estimation result after 5 % truncation of the sample, with an estimated coefficient of 0.0033 for agricultural subsidies, which is significant at the 10 % statistical level. Model (9) is the estimated result after 5 % truncation of the sample, with an estimated coefficient of 0.0045 for agricultural subsidies, which is significant at the 5 % statistical level. This indicates that after adjusting the sample, agricultural subsidies still significantly improve the technical efficiency of rapeseed production, indicating that the results of the benchmark regression are robust.

**Table 7:** The results of adjusting sample.

Variables	Model (8)		Model (9)	
	Coef.	SE	Coef.	SE
*Subsidy*	0.0033*	(0.0020)	0.0045**	(0.0021)
*Gender*	−0.0272	(0.0232)	−0.0203	(0.0235)
*Age*	0.0001	(0.0007)	0.0002	(0.0007)
*education*	−0.0317***	(0.0090)	−0.0325***	(0.0091)
*Health*	0.0196*	(0.0108)	0.0255**	(0.0109)
*Family*	−0.0063*	(0.0034)	−0.0066*	(0.0034)
*farming*	0.0013	(0.0074)	0.0020	(0.0076)
*Traffic*	0.0012	(0.0017)	0.0020	(0.0017)
*cooperative*	−0.0083	(0.0139)	0.0041	(0.0143)
*training*	0.0201	(0.0135)	0.0175	(0.0134)
*machine*	0.0169	(0.0162)	0.0142	(0.0169)
*information*	−0.0317*	(0.0175)	−0.0280	(0.0173)
*Constant*	0.7884***	(0.0683)	0.7505***	(0.0694)
*Observations*	609		578	

### 3.5 Results of threshold regression

A threshold regression model was used to further investigate the effect of agricultural subsidies on the technical efficiency of various planting area. Table 8 shows that single threshold model is significant at the 1 % statistical level, indicating that agricultural subsidies have the single threshold effect on the technical efficiency. The double threshold model, on the other hand, failed the significance test, showing that there is no double threshold. Table 9 shows the estimation results after dividing the sample into two intervals based on the planting area of the threshold.

**Table 8:** Results of the threshold effect tests for planting area.

Model	Single and double threshold test results					
	BS’ number of times	F value	P value	1 %	5 %	10 %
Single threshold	500	9.815***	0.002	8.172	4.204	2.988
Double threshold	500	1.728	0.207	5.942	4.410	2.883

**Table 9:** Results regarding agricultural subsidies and technical efficiency using the threshold model.

Variables	Model (10)		Model (11)	
	Coef.	SE	Coef.	SE
*subsidy*	0.0101***	(0.0027)	0.0009	(0.0037)
*gender*	−0.0218	(0.0272)	−0.0736	(0.0715)
*age*	−0.0005	(0.0009)	0.0011	(0.0022)
*education*	−0.0476***	(0.0110)	0.0143	(0.0230)
*health*	0.0219*	(0.0130)	0.0853***	(0.0305)
*family*	−0.0096**	(0.0040)	−0.0162	(0.0100)
*farming*	−0.0002	(0.0091)	0.0282	(0.0190)
*traffic*	0.0037*	(0.0021)	0.0071	(0.0048)
*cooperative*	0.0160	(0.0187)	−0.0077	(0.0295)
*training*	0.0229	(0.0157)	0.0834*	(0.0458)
*machine*	0.0563**	(0.0222)	−0.0079	(0.0354)
*information*	−0.0311	(0.0195)	0.1066	(0.0908)
Constant	0.8304***	(0.0832)	0.3113*	(0.1875)
Observations	461		148	

In Table 9, model (10) is estimated for operation size below the threshold (*land* < 0.67). It is revealed that the coefficient of *subsidy* is positive and significant at the 1 % statistical level when planting area is less than 0.67 ha, indicating that agricultural subsidies significantly improve the technical efficiency. Model (11) is the estimation result for planting area above the threshold (*land* ≥ 0.67). This further reveals that the coefficient of *subsidy* remains positive but statistically insignificant when planting area is larger than 0.67 ha, indicating that the effect of agricultural subsidies on improving the technical efficiency is not significant. The reason could be that for small farmers whose planting area is less than 0.67 ha, agricultural subsidies can alleviate financial constraints and can encourage small farmers to adopt more advanced technology and management, thus improving production efficiency. As for large farmers with more than 0.67 ha, they have certain financial advantages, and the effect of agricultural subsidies encourage them to improve their technical efficiency when it was declining. In addition, among the control variables, when planting area is less than 0.67 ha, the education level of farmers significantly reduces the technical efficiency, while the role of agricultural training on technical efficiency is not significant. However, when planting area is larger than 0.67 ha, the coefficient of *education* is positive, and agricultural training significantly increases technical efficiency. This implies that the role of human capital in improving technical efficiency is more significant when the planting area is expanding.

## 4 Discussion

This study finds that agricultural subsidies have a significant positive impact on technical efficiency, which contradicts with the findings of Bongfiglio et al., 2020, Fyrd et al., 2021, and Minviel et al., 2017, who discovered that agricultural subsidies have negative impact on technical efficiency [24,44,45]. This is worth noting that the impact of agricultural subsidies on technical efficiency varies according to the level of the country's economy [46]. It is evident from the literature that sample data for these studies all belong to developed countries. They generally believe that the motivation of farmers to work efficiently is lower when they depend to a higher degree on subsidies as a source of income [47]. And agricultural subsidies might skew resource allocation efficiency and dampen farmers' production enthusiasm. In China, the development of the rural financial market is still not mature enough, and farmers have insufficient access to capital [48]. Thus, agricultural subsidies, as a policy-based financial instrument to support agricultural development, can give relief to farmers' financing constraints, increase the likelihood that farmers will have timely access to the input factors they need and improve their production techniques, which may lead to an increase in technical efficiency. In other words, agricultural subsidies become a source of finance for farmers, enabling them to produce successfully. At the same time, when farmers become more efficient in their production, they become more profitable and receive more agricultural subsidies, which can also provide an incentive for farmers to improve their technical efficiency [49].

Moreover, some literature argues that agricultural subsidies do not ensure food security and sustainable production [50]. Therefore, the ability of agricultural subsidies to achieve some of the policy objectives is a matter of concern. We discovered that agricultural subsidies have a significant positive impact on rapeseed planting area, which is consistent with the findings of Ganbold et al., 2022 and Si et al., 2023 [51,52]. In China, the problem of land fragmentation has been an important constraint to the modernization of agricultural production [53]. From this perspective, this research reveals that agricultural subsidies have increased farmers' financial resources, enabling them to expand the scale of their operations, which suggests that policy objectives of subsidies for large-scale operations have been realized in China [54].

Our findings also show that agricultural subsidies significantly reduce the use of chemical fertilizers. This is consistent with the findings of Guo et., 2021 [41]. In China, the overuse of chemical fertilizers has led to poor soil quality and environmental pollution, which is not conducive to sustainable agricultural development [55]. Agricultural subsidies can motivate farmers to make long-term investments [56], alleviate China's excessive use of fertilizers, and thus have a positive impact on green production [57]. This study suggests that the policy objective of arable land conservation subsidies is being realized in China. Therefore, there is a significant need for green production subsidies in China for the cultivation of rapeseed oil. To encourage farmers to produce sustainably, we propose that the current Chinese subsidy model be further subdivided, for example, by subdividing the arable land fertility subsidy into green pesticide subsidy, green fertilizer subsidy, and fallow subsidy, among others.

### 4.1 Conclusion and policy implications

This research collected data from 609 field yield measurements from the National Rapeseed Oil Industrial Technology System in 2020 to analyze the effects of agricultural subsidies on technical efficiency and factor inputs by using a stochastic frontier production function, instrumental variables method, and threshold regression models. The main findings revealed: 1) Using instrumental variables to control for endogeneity and the one-step SFA approach, the results show that agricultural subsidies increase the technical efficiency; 2) Agricultural subsidies have positive impact on land inputs and negative impact on fertilizer inputs, but they do not have significant effects on labor and capital inputs either; 3) Agriculture subsidies have a threshold influence on technical efficiency. Agricultural subsidies have greater impact on the technical efficiency of farmers whose planting area is less than 0.67 ha, but they do not have the same effect on farmers whose planting area is greater than 0.67 ha.

The aforementioned study results have three policy implications. The first is to increase the total amount of subsidies while simultaneously increasing investments in agricultural assistance and protection subsidies. For the purpose of stabilizing the expectations of farmers regarding agricultural production and making full use of the policy effect of agricultural subsidies in promoting technical efficiency, it is recommended that the fundamental structure of agricultural subsidy policy be maintained while the intensity of subsidies be expanded. Secondly, we need to speed up the creation of a green production-oriented subsidy system and expand the types of subsidies available. We recommend global support for innovative subsidy models that teach farmers to use fertilizer and pesticides more scientifically, make the maximum of agricultural subsidies' role in promoting green output, and speed up the shift to intensive farming practices. Finally, we must optimize the subsidy system and adopt a subsidy plan that is more flexible. It has been proposed that subsidies be increased for low-income households that operate small businesses, and that in addition, these households should be encouraged to adopt innovations in production techniques. Finally, subsidies to assist agricultural skill training should be considered in policy framework, as one subsidy approach might not be enough to improve the technological efficiency for large-scale households.

### 4.2 Further research

This research analyzed the impact of agricultural subsidies on the technical efficiency of rapeseed production in China. However, it's important to acknowledge two main limitations within this research. Firstly, the cross-sectional data used in this study cannot address unobservable individual heterogeneity characteristics during data processing. In the future, research can continuously observe individual farmers and collect data for more years. Secondly, this study used a summary of four types of subsidies (scale management subsidies, machinery subsidies, arable land conservation subsidies, and other subsidies), and insufficient analysis was conducted on the effects of different types of subsidies. In the future, research can clarify the different impacts that various subsidies have on technical efficiency of the rapeseed in China.

## Funding statement

This research is supported by the China Agriculture Research Systems (CARS-0012); National Natural Science Foundation Youth Program (71703048).

## Data availability statement

This paper's dataset and model estimation codes are available at the Mendeley data repository and can be accessed at following citation: Shahzad, Muhammad Aamir (2024), “An analysis of the effect of agriculture subsidies on technical efficiency: Evidence from rapeseed production in China”, Mendeley Data, V1, https://doi.org/10.17632/vbrchjk3fd.1.

## CRediT authorship contribution statement

**Fuxing Liu:** Writing – original draft, Data curation, Conceptualization. **Muhammad Aamir Shahzad:** Writing – review & editing, Resources, Project administration, Methodology, Investigation, Formal analysis, Conceptualization. **Zhongchao Feng:** Writing – review & editing, Resources, Funding acquisition. **Lianfen Wang:** Supervision, Software, Project administration, Investigation, Funding acquisition. **Juan He:** Writing – original draft, Visualization, Validation, Software, Methodology.

## Declaration of competing interest

The authors declare that they have no known competing financial interests or personal relationships that could have appeared to influence the work reported in this paper.
