# Supplementary material for: An analysis of the effect of agriculture subsidies on technical efficiency: Evidence from rapeseed production in China
Source: Heliyon. 2024 Jun 28;10(13):e33819. doi: 10.1016/j.heliyon.2024.e33819 (PMC11263720; doi:10.1016/j.heliyon.2024.e33819)
Supplement: Multimedia component 1 [file mmc1.doc]

**Study Questionnaire**

Survey Form for Farmers on the Current Situation of Rape Production (Winter Rape Production Area) Investigator Date Phone number

**Informed Consent Statement**

I consent to participate in the research project and the following has been explained to me: the research may not be of direct benefit to me. my participation is completely voluntary. → Next section

**Part A. BASIC INFORMATION OF FARMERS**

(1) Home address: province county village

(2) Name of household head: Name of production decision-maker:

(3) Gender : □ Male □ Female

(4) Date of birth:

(5) Years of Education:

(6) Health condition: □ Good □ Same □ Bad

(7) Household population: Labor force quantity: Number of migrant workers:

(8) Is it a party member or village cadre household: □ Yes □ No

(9) Distance to the nearest farmers' market (km):

(10) Distance to nearest agricultural technology promotion institution (km):

**Part B. BASIC DATA OF RAPESEED PRODUCTION**

| **Basic data of rapeseed production** | **2019-2020** |
| --- | --- |
| Planting area of rapeseed (ha) |  |
| Among them, machine sowing area (ha) |  |
| Rapeseed machine cultivated area (ha) |  |
| Seed consumption of rapeseed (catties per household) |  |
| The name of the rapeseed variety used |  |
| Fertilizer application amount for rapeseed (catties per household) |  |
| Input of rapeseed labor force (worker/household) |  |
| Among them, employee input (per household) |  |
| Harvest date of rapeseed (Gregorian calendar: MM/DD/YYYY) |  |
| Harvest area of rapeseed (ha) |  |
| Among them, machine harvested area (ha) |  |
| Loss rate of rapeseed harvest (%) |  |
| Affected area of rapeseed (ha) |  |
| Total rapeseed production (per kilogram) |  |
| Oil yield of rapeseed (%) |  |
| Sales quantity of rapeseed (catties) |  |
| Household consumption of edible oil (per kilogram) |  |
| Agricultural machinery subsidies in place (yuan) |  |
| Subsidy for large-scale operation in place (yuan) |  |
| Subsidy for land fertility protection in place (yuan) |  |
| Other subsidies in place (yuan) |  |

**Part C. FARMERS' ECONOMY, ATTITUDES, AND NEEDS**

| **Farmers' Economy, Attitudes, and Needs** | **2019-2020** |
| --- | --- |
| Land transfer price (yuan/mu/year, average price for non-transfer) |  |
| Input of rapeseed seeds (yuan, discounted from retained seeds) |  |
| Input of rapeseed fertilizer (including fertilizer and farmhouse fertilizer) (yuan) |  |
| Agricultural labor price (yuan/worker, average price for unemployed workers) |  |
| Cost of rapeseed machinery operation (yuan) |  |
| Other inputs (such as pesticides, agricultural film, water and electricity, etc.) (yuan) |  |
| Number of rural labor force at home (person) |  |
| Sales price of rapeseed (yuan/kg, average price for unsold items) |  |
| Sales revenue of rapeseed (including seeds, oil, and meal) (yuan) |  |
| Household agricultural income (yuan) |  |
| Income from family work (yuan) |  |
| Other household income (yuan) |  |
| Disaster species (A drought, B waterlogging, C freezing, D insect, E disease, F grass, G no) |  |
| Household food expenditure (yuan) |  |
| Household agricultural expenditure (yuan) |  |
| Other household expenses (yuan) |  |
| When bolting, the area with plucked bolts (ha) |  |
| During the flowering season, do beekeepers come to the fields to gather honey？(yes or no) |  |
| Is there anyone coming for sightseeing during the flowering season？(yes or no) |  |
| Is there anyone around who tills and presses rapeseed for green manure？(yes or no) |  |
| Is there anyone around who uses rapeseed as feed and sells it？(yes or no) |  |

**Part D. PRODUCTION WILLYING AND SOCIOL ECONOMIC CONDITIONS**

(1) Your family plans to plant an area of acres of rapeseed for the next season: ha

(2) The minimum purchase price you are willing to accept for rapeseed: yuan/kg

(3) What is the impact of the following traits on your selection of rapeseed varieties? (Degree: 1. Very low; 2. Low; 3. Average; High; 5. Very high)

**Your choose:** □ Seed price □ Insect resistance □ Disease resistance □ Anti lodging □ Germination rate □ Double low □ Oil yield □ Yield □ Suitable for mechanization

(4) Degree of satisfaction with one's own relevant situation (with the same level as the previous question):

**Your choose:** □ Production of rapeseed □ Agricultural production □ Overall living conditions

(5) Is there an agricultural cooperative (cooperative, rapeseed professional association, etc.) organization nearby:

**Your choose:** □ No □ Yes.

Have you joined it?

**Your choose:** □ No □ Yes

(6) Is your home a family farm recognized by the local government?

**Your choose:** □ No □ Yes

(7) This year, the number of households receiving agricultural technology training, total number of days :

Does anyone at home hold various agricultural qualification certificates:

**Your choose:** □ Yes □ No

(8) Do you have the following tools at home:

**Your choose:** □ TV □ landline phone □ mobile phone without internet access □ mobile phone with internet access □ WeChat □ computer

(9) Major disasters during 2019 to 2020

**Your choose:** □ Drought □Waterlogging □Frost damage □ Insect damage □Disease □ Grass damage □ No major disasters

(10) What is the impact of the following factors on the decision of your family's rapeseed planting area for the next season?

**Your choose:** □ Improved seed policy □ Sales price of rapeseed □ Cost of means of production □ Labor input □ Climate environment □ Crop contradiction

(11) When the cumulative subsidy amount for rapeseed reaches_____ Willing to increase planting area at a rate of yuan/kg; If there is no subsidy and storage policy for rapeseed, we are willing to maintain the planting area of_____ ha.

(12) What form of rapeseed seed subsidy would you prefer to accept?

**Your choose:** □Free or low-priced government planting □Planting varieties from the recommended catalog and receiving cash

(13) Do you understand the target price subsidy policy:

**Your choose:** □ Yes □ No

And would prefer to accept the subsidy calculation method

**Your choose:** □Supplement by sales volume □ Supplement by production □ Supplement by area

(14) Is there a health clinic

**Your choose:** □ Yes □ No

primary school:

**Your choose:** □ Yes □ No

hardened roads:

**Your choose:** □ Yes □ No

and running water:

**Your choose:** □ Yes □ No

**NOTE:**

1. The start and end times of the questionnaire are consistent with the growth cycle of rapeseed, with a statistical period from June 1st of the previous year to May 30th of the following year.

2. The "production decision-maker" referred to in the questionnaire refers to the individual who currently determines the various production behaviors in agricultural production of the household. The questionnaire should try to inquire with this person, and the agricultural production decision-maker may not be the head of the household.

3. The unit of area in this questionnaire is "mu", 1 mu=666.67 square meters; The unit of mass is "jin", where 1 jin=0.5 kilograms=500 grams; The unit of labor is "work", which refers to the amount of labor input by producers (including their family members and hired workers) during the rapeseed production process. One "work" refers to a labor force working for one day, and working for 8 hours is considered a day.

4. The "existing arable land area" refers to the total area of arable land that the household currently has the right to use (operate), and the "existing contracted area" refers to the total area of arable land that the household currently has the right to contract in the local area. Typically, the "existing arable land area=existing contracted land transfer in area - land transfer out area".

5. Land transfer "refers to the act of a farmer transferring the use right (i.e. management right) of the contracted land to other farmers or economic organizations for agricultural production. Land acquisition and other actions that change the use of land do not belong to land transfer. If the household has not experienced land transfer, fill in the local average for the data of "land transfer price".

6. 'Winter fallow field' refers to the area of land in the household's existing farmland that has not been planted with winter crops, while 'abandoned area' refers to the area of land in the household's existing farmland that has not been planted with any crops for more than a year.

7. When conducting a geographical location survey, if the household has multiple plots of land, priority should be given to filling in the geographical location of the surveyed plots. If there are multiple plots of land, fill in the geographical location of the largest plot of land. If it is convenient for statistics, please measure in the middle of the plot. Fill in degrees, minutes, and seconds for longitude and latitude indicators, and meters for altitude indicators.

8. In this questionnaire, unless otherwise specified, the input-output statistics in rapeseed production are based on the total input and output of the household, not the input-output per unit area.

9. The "affected area" refers to the planting area where the actual harvest of crops is reduced by more than 10% compared to the annual yield due to drought, floods, wind and hail disasters, frost, pests and diseases, and other natural disasters.

10. The farm manure used by farmers in rapeseed production, if produced by themselves, will be discounted based on the local average sales price of this type of farm manure and included in the "input of rapeseed farm manure" column.

11. If the household has not sold rapeseed and rapeseed oil this year, please inquire about the local average selling price of rapeseed and rapeseed oil in the columns of "rapeseed sales price" and "rapeseed oil sales price".

12. The "scale operation subsidy" refers to the agricultural subsidy that can only be received by large farmers, family farms, and other farmers who have reached a certain scale of arable land in the local area.

13. The amount in the "food expenditure" column includes the cash value of the farmer's self-produced and self-used agricultural products, calculated at the local average price.

14. The options in question 3 and question 4 are independent of each other and are not directly sorted. Each number can be used multiple times.

15. In the column of oil extraction fees in question 12, if the oil extraction workshop does not charge fees for retaining rapeseed meal, record a value of 0; If the oil mill returns a certain amount of cash to farmers for retaining rapeseed meal, this item is recorded as a negative value based on the payment price.

16. In the first question of question 14, if the option for farmers is' no ', the investigator should fully explain the detailed information of the target price subsidy policy before asking the next question.

17.The target price policy is an agricultural support policy that protects the interests of producers through price difference subsidies based on the formation of agricultural product prices in the market. After implementing the target price policy, the temporary purchase and storage policy will be canceled, and producers will sell agricultural products at market prices. When the market price is lower than the target price, the state will calculate the amount of subsidies that should be granted according to the difference between the target price and the market price, and the factors such as planting area, output or sales volume, and give subsidies to producers; When the market price is higher than the target price, the state will not grant subsidies.
